# Supplementary material for: Time Course of Changes in the Neurovascular Unit after Hypoxic-Ischemic Injury in Neonatal Rats
Source: Int J Mol Sci. 2022 Apr 10;23(8):4180. doi: 10.3390/ijms23084180 (PMC9027443; doi:10.3390/ijms23084180)
Supplement: Supplementary file 1 [file ijms-23-04180-s001.zip › ijms-1614978-supplementary.pdf]

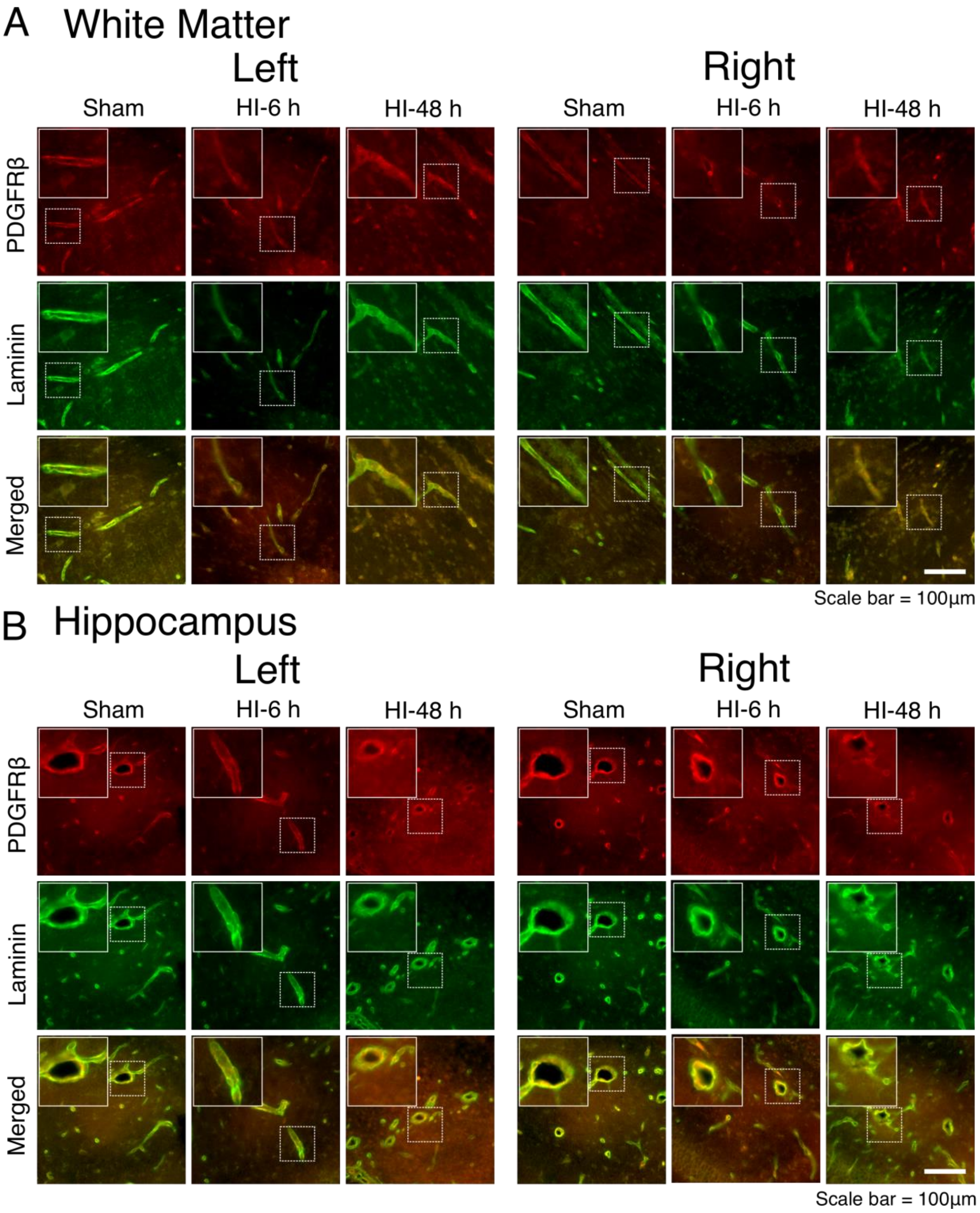

Figure S1. Immunohistochemical expression of pericyte coverage in white matter, and hippocampus on the left hypoxic and right HI sides in the brain of the neonatal rats in the Sham, HI-6 h and HI-48 h groups. (**A**) Representative images of pericytes (PDGFRβ, Red), microvessels (Laminin, Green) and merged double immunostaining in white matter of left hypoxic and right HI sides of rat brain in Sham, HI-6 h, and HI-48 h. Magnification, 40×. Each inset contains high magnification images. Scale bar = 100 µm. (**B**) Representative images of pericytes (PDGFRβ ,Red), microvessels (Laminin, Green) and merged double immunostaining in hippocampus of left hypoxic and right HI sides of rat brain in Sham, HI-6 h, and HI-48 h. Magnification, 40×. Each inset contains high magnification images. Scale bar = 100 µm.

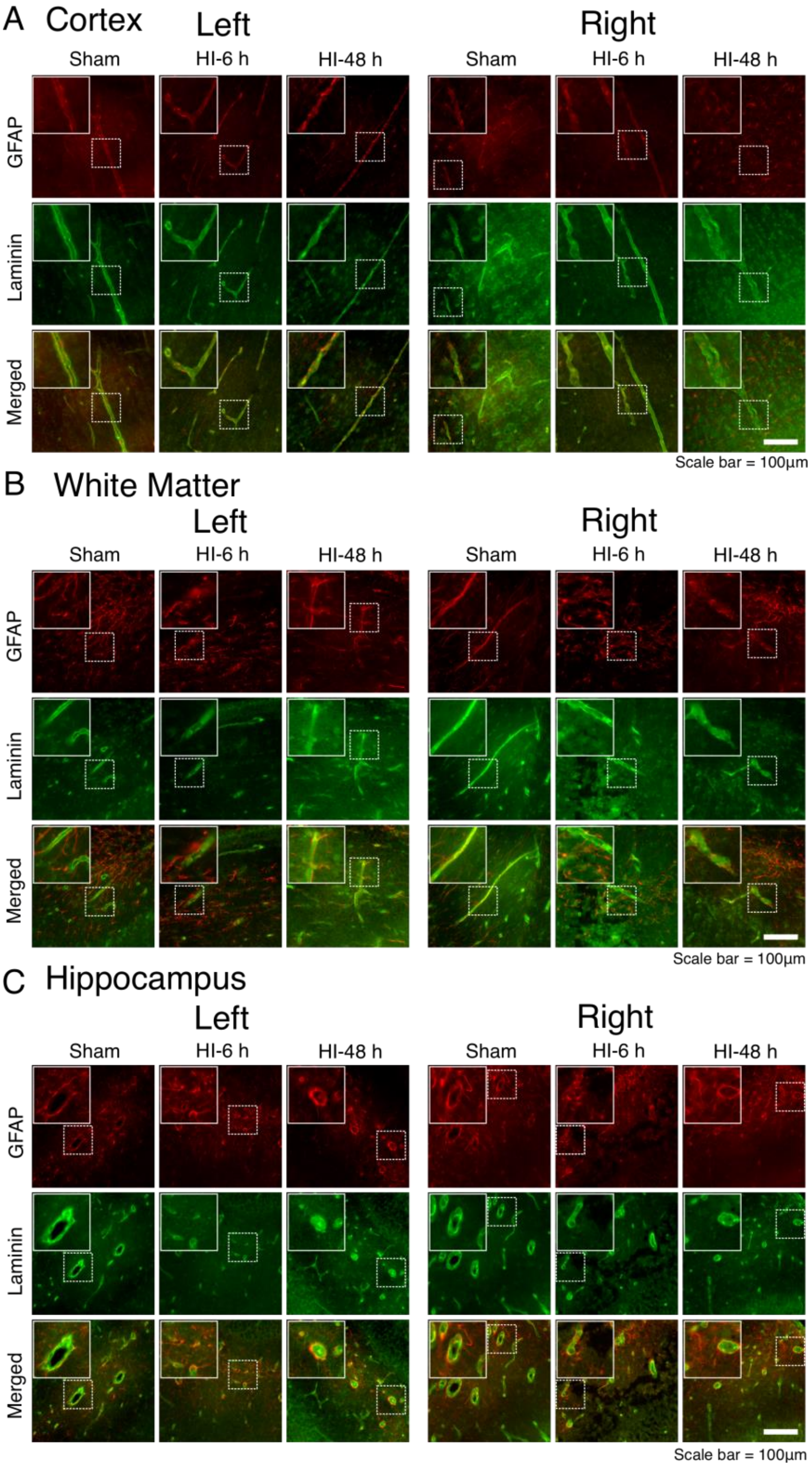

Figure S2. Immunohistochemical expression of astrocyte coverage in white matter, and hippocampus on the left hypoxic and right HI sides in the brain of the neonatal rats in the Sham, HI-6 h and HI-48 h groups. (**A**) Representative images of astrocytes (GFAP, Red), microvessels (Green) and merged double immunostaining in cerebral cortex of left hypoxic and right HI sides of rat brain in Sham, HI-6 h, and HI-48 h. Magnification, 40×. Each inset contains high magnification images. Scale bar = 100 μm. (**B**) Representative images of astrocytes (GFAP, Red), microvessels (Green) and merged double immunostaining in white matter of left hypoxic and right HI sides of rat brain in Sham, HI-6 h, and HI-48 h. Magnification, 40×. Each inset contains high magnification images. Scale bar = 100 μm. (**C**) Representative images of astrocytes (GFAP, Red), microvessels (Green) and merged double immunostaining in hippocampus of left hypoxic and right HI sides of rat brain in Sham, HI-6 h, and HI-48 h. Magnification, 40×. Each inset contains high magnification images. Scale bar = 100 μm.

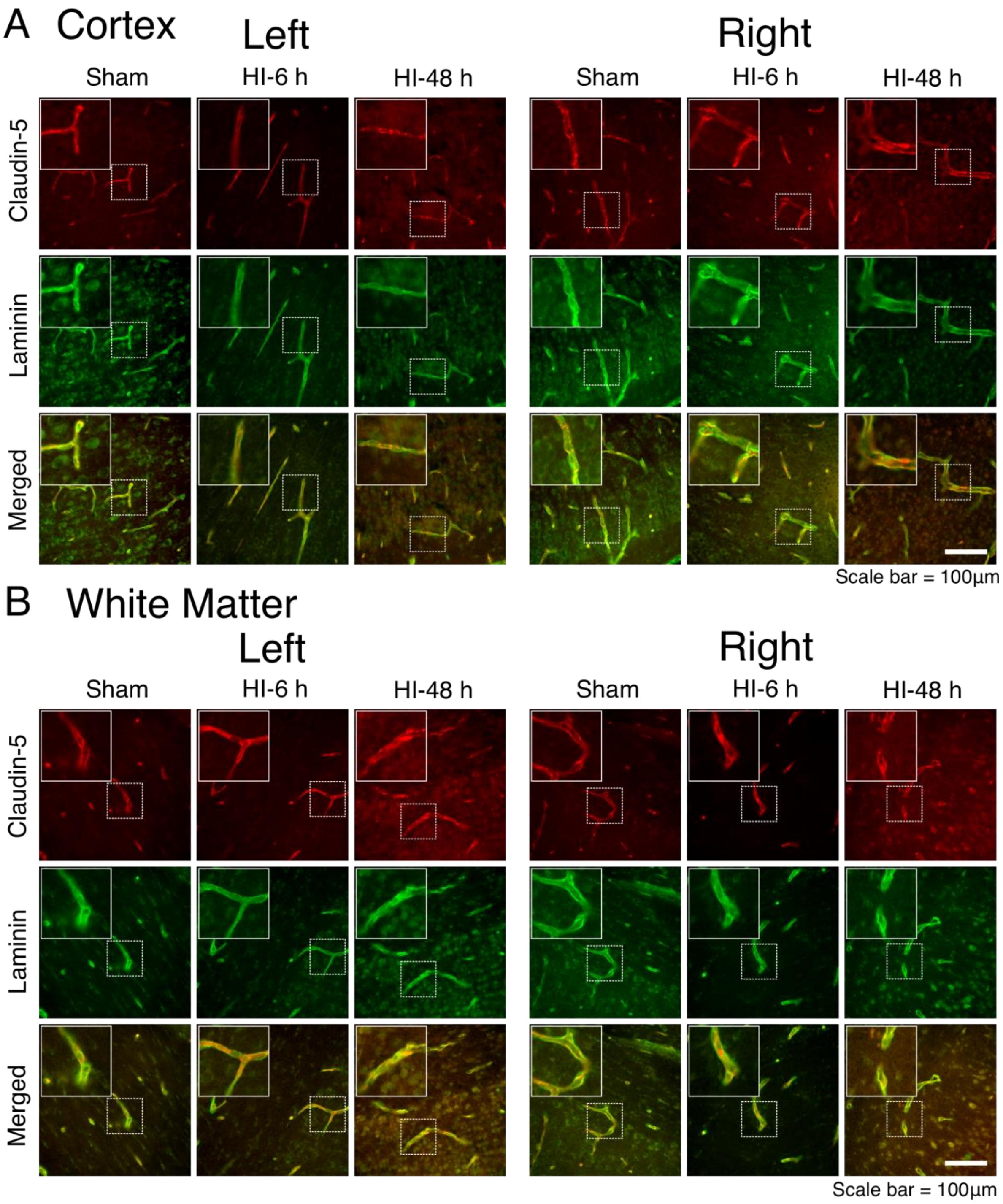

Figure S3. (**A**) Representative images of claudin-5 (Claudin-5, Red), microvessels (Laminin, Green) and merged double immunostaining in cerebral cortex of left hypoxic and right HI sides of rat brain in Sham, HI-6 h, and HI-48 h. Magnification, 40×. Each inset contains high magnification images. Scale bar = 100 μm. (**B**) Representative images of claudin-5 (Claudin-5, Red), microvessels (Laminin, Green) and merged double immunostaining in white matter of left hypoxic and right HI sides of rat brain in Sham, HI-6 h, and HI-48 h. Magnification, 40×. Each inset contains high magnification images. Scale bar = 100 μm.
